# Conservative management versus volar plating for dorsally displaced distal radius fractures in the elderly: A randomized control trial

**DOI:** 10.12669/pjms.39.3.6522

**Published:** 2023

**Authors:** Saeed Ahmed Shaikh, Muhammad Tahir, Nadeem Ahmed, Mauro Maniglio

**Affiliations:** 1 Dr. Saeed A. Shaikh, FCPS. Department of Orthopaedics, Surgical Building, Jinnah Postgraduate Medical Centre, Rafiqui Shaheed Road, Karachi, Pakistan; 2 Dr. Muhammad Tahir, FCPS. Department of Orthopaedics, Surgical Building, Jinnah Postgraduate Medical Centre, Rafiqui Shaheed Road, Karachi, Pakistan; 3 Dr. Nadeem Ahmed, FCPS. Department of Orthopaedics, Surgical Building, Jinnah Postgraduate Medical Centre, Rafiqui Shaheed Road, Karachi, Pakistan; 4 Dr. Mauro Maniglio, MD. Department of Hand and Plastic Surgery, CHUV, University Hospital Lausanne, Switzerland

**Keywords:** Distal radius fracture, Colle’s fracture, Volar plating, Plaster immobilization

## Abstract

**Objective:**

This randomized trial aims to compare the clinical, and radiological outcomes between plaster cast and volar plating for distal radius fractures (DRF) in the elderly at six months, and one-year.

**Methods:**

A randomized trial was performed at Jinnah Postgraduate Medical Centre between February 2015 and April 2020. The study included patients that were above 60 years but under 75 with an isolated, closed, unilateral, dorsally displaced DRF. Randomization into two groups (casting or plating) was based on a computer-generated algorithm stratified by age group and AO/OTA fracture type. The primary outcome was Patient Rated Wrist Evaluation score. Secondary clinical outcomes were active range of motion, grip strength, the Mayo’s wrist score and the quick Disability Arm, Shoulder, Hands scale. Patient’s satisfaction was evaluated with use of a SF-12 questionnaire and finally complications were recorded.

**Results:**

This trial has shown that there is no significant difference in clinical outcomes of DRF at six and twelve months follow up when treated by cast immobilization or plating. Although, the radiological parameters and the number of complications were significantly higher in the immobilization group

**Conclusion:**

The results of the trial have shown that plating and casting are equally effective in achieving satisfactory patient reported and clinical outcomes at intermediate and final follow-up restoring patient satisfaction.

***Trial registration:*** The trial is registered in the Chinese Clinical Trial Registry. The trial registration number is ChiCTR2000032843, and the URL is: http://www.chictr.org.cn/searchprojen.aspx

## INTRODUCTION

As per Lafontaine’s criteria age over 60 years is an indicator of unstable distal radius fracture (DRF)^1^ which can re-displace despite closed manipulation and reduction (CMR).^2^ Therefore, the trend is towards volar locking plate fixation^3^ due to good long-term results.^4^ In addition, published literature quotes superior restoration of the bony anatomy with volar locking plates.^5^,^6^ However, achieving better radiographic parameters does not translate into better functional outcomes.^7^,^8^ Furthermore, elderly patients are able to cope better malunited DRFs.^9^

As a result, there is variability in the published literature which does not guide us on the choice of operative or conservative treatment of DRF^9^ in the elderly population.^7^,^10^,^11^ Therefore, the authors aimed to perform a RCT comparing cast immobilization with open reduction and internal fixation (ORIF) in patient between 60-75 years with dorsally displaced DRFs, with the hypothesis that ORIF leads to a better clinical outcome, mainly in the Patient Rated Wrist Evaluation score (PRWE) at one-year.

## METHODS

### Inclusion and exclusion criteria:

The inclusion criteria for recruitment to trial were patients aged between 60-75 years with a closed, isolated dorsally displaced DRF (intra-articular or extra-articular) AO/OTA 2R3 A2, A3 or C1-C3 types^12^ presented within seven days of injury. The following patients were excluded from the trial:

1. Bilateral DRF.
2. Open fracture.
3. Fracture extending more than 3 cm from the radiocarpal joint.
4. Ipsilateral limb fractures/injuries, multiple injuries or polytrauma patients.
5. Previous fracture of the hand or wrist.
6. Impaired wrist function secondary to arthritis/ rheumatoid hands/ malunited DRF.
7. Patients unfit for anesthesia.
8. Patients lacking capacity due to poor cognition i.e., previous history of stroke or dementia

### Randomization:

Randomization was done, after patients’ consent on 1:1 basis stratified by fracture type based on a computer-generated randomization code. An independent team was allocated to assess the outcomes of the study.

### Cast immobilization group:

Patients randomized to cast immobilization were initially treated in emergency department with CMR under a hematoma block^13^ and subsequently a backslab was applied which was converted to a complete plaster after two-weeks. The cast was removed after radiographic signs of callus formation and clinically absence of pain on wrist movement. Patients in the cast immobilization group were advised to move their fingers and squeeze a ball during the time of immobilization. After cast removal full range of movement was allowed.

### Open reduction internal fixation group:

For the operative group, surgery was performed through a Henry approach with fracture fixation done via volar locking plate (Double Medical, Fujian, China) under fluoroscopic guidance The choice of anesthesia was depends on the surgeon, and was general, regional nerve block or wide-awake local anesthesia (WALANT). Postoperatively, no splint was applied, and patients were instructed to move the wrist as pain allowed within the first six weeks without weight-bearing. Rehabilitation was started under supervision of a physiotherapist and weightbearing was started at the discretion of the treating surgeon.

### Outcomes:

Three, six, and twelve months were reported. Patient-rated wrist evaluation (PRWE)^14^ score at 12 months was the primary outcome. Zero on the PRWE indicates no pain or functional impairment. The PRWE minimal clinically important difference (MCID) for trauma was defined at 11.5^15^ according to the calculations of Walenkamp et al.^15^ The secondary outcomes were the Mayo wrist score from 0 to 100, with 100 indicating full function, and the quick Disability Arm, Shoulder, Hands (qDASH) score from 0 to 100, with 0 indicating no disability.

Quality-of-life assessment using the patient satisfaction form (SF-12); wrist flexion, extension, pronation, and supination measured with a goniometer; and grip strength measured with a dynamometer and reported as a percentage of the contralateral uninjured wrist. After treatment, radiographs showed palmar/dorsal tilt, radial inclination, ulnar variance, and intra-articular step-off. Both groups had twelve-weeks, six-months, and twelve-month radiographs. On anteroposterior and lateral views, fracture union was defined as radial, ulnar, and dorsal cortical bone bridging. The complications noted were loss of reduction, fracture malunion, fracture non-union, deep infection, neuropathy, tendon irritation, tendon rupture, dysesthesia and hyperesthesia in the injured hand, vasomotor changes, skin atrophy, and diffuse osteopenia^16^ diagnosed as CRPS.

### Sample-size calculation:

Sample size was calculated prior to the study based on the primary outcome. The minimum clinical difference for DRF in the PRWE score was set at 11.5^15^ as explained above. We had assumed an alpha error of 0.05 and an allocation of 1:1 based on BMI and fracture type. At 95% of power, the sample size was 324. Owing to an expected 10% participant to a loss of follow-up, we increased our sample size to 378.

### Data analysis:

The continuous data was reported as mean and standard deviation or range whereas the categorical data was reported as frequencies and percentages. A t-test for independent samples or a nonparametric Mann-Whitney U test was performed for the determination of differences of mean values between the two treatment groups. Chi-square test and Fischer exact test was performed for the categorical data. Statistical analysis was performed in SPSS version 22.6 (IBM Corp., Armonk, N.Y., USA). The p-value was set at 0.05 with a confidence interval of 95%.

### Ethical approval:

With the approval of ethics review committee of Jinnah Postgraduate Medical Centre, Karachi (NO. F .2-81/2015-GEN L/1711/JPMC) a single center parallel design RCT was conducted between February 2015 and April 2020.

## RESULTS

**Fig.1:**
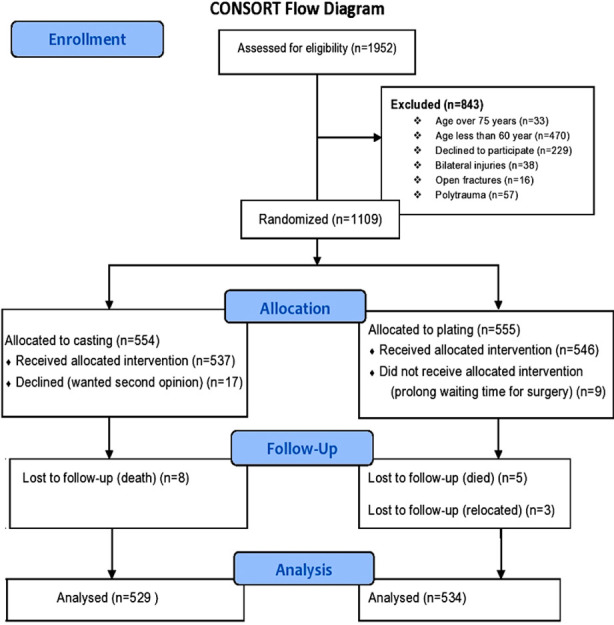
CONSORT flow diagram.

Between August 2016 and December 2019, 3092 patients were screened for eligibility out of which 28.42% (1109/3902) patients fulfilled the inclusion criteria. In the casting group 554 patients were recruited and 555 were assigned to the plating group. The lost to follow-up in the casting and in the plating group were eight each respectively as shown in Fig.1. The mean age in our study was 66 years and 62% of the patients were females. The baseline characteristics of the two groups were well balanced as suggested by p-values in (Table-I).

**Table-I:** Comparison of Baseline Variables among Patients in Both Groups.

Variable	Cast-Immobilization (529)	Volar Locking Plate (534)	p-value
Mean age in years	65.16±3.90	64.90 ±3.70	0.25
***Gender (percentage)***			0.66
Females	330 (62.4)	326 (61.0)	
Males	199 (39.0)	208 (37.6)	
***Injured wrist (percentage)***			0.14
Dominant	327 (61.8)	354 (66.3)	
Non-dominant	202 (38.2)	180 (33.7)	
***Type of Fracture (percentage)***			0.81
Extra-articular	246 (46.5)	244 (45.7)	
Intra-articular	283 (53.5)	290 (54.3)	
***AO/OTA Classification (percentage)***			0.05
A2	182 (34.4)	189 (35.4)	
A3	64 (12.1)	55 (10.3)	
C1	204 (38.6)	178 (33.3)	
C2	57 (10.8)	73 (13.7)	
C3	22 (4.2)	39 (7.3)	
Mean time required for Fracture Union in weeks	15.34 ±2.18	15.34± 2.26	0.74
Mean BMI (kg/m^2^)	26.48 ±3.47	26.65 ±3.75	0.48
Mean Height in meters	1.71±0.052	1.71 ±0.056	0.16
Mean Weight in kilograms	77.73±10.08	77.79 ±10.68	0.93
***BMI group (percentage)***			0.11
Under 30 kg/m^2^	441 (83.4)	425 (79.6)	
Above 30 kg/m^2^	88(16.6)	109 (20.4)	
***Number of Comorbid conditions (percentage)***			0.01
Less than 2	233 (44.0)	276 (51.7)	
More than 2	296 (56.0)	258 (48.3)	
***ASA grade (percentage)***			0.16
II	40 (7.6)	56 (10.5)	
III	428 (80.9)	409 (76.6)	
IV	61 (11.5)	69 (12.9)	

1. p-value for Categorical variable was calculated by Chi-square

2. p-value for continuous variable was calculated by independent t-test.

At three months follow up plating group had significantly better functional outcomes in comparison to plaster group, although there was no statistical difference in patient satisfaction as shown by their PRWE, qDASH and Mayo’s wrist scores in comparison to cast group, although there determined by SF-12 scores. However, at six and 12 months follow up, there was no significant difference in the functional outcome scores between the two groups suggesting that both interventions were equally effective in our study population. At various intervals of follow-up during the trial, there was no significant difference in range of movement or grip strength (Table-II).

**Table-II:** Patient Reported Outcomes (PROMS) of the two treatment groups at three months, six months, and one-year.

PROMS	Cast-Immobilization (529)	Volar Locking Plate (534)	P=value
***3 months***			
PRWE score	44.65 ±2.34	45.12 ±4.42	0.48
qDASH score	23.59 ±9.70	20.58 ±9.76	0.001
Mayo’s wrist score	43.21 ±7.96	38.28 ±10.15	0.001
SF-12 (PCS)	30.19± 5.05	30.83 ±5.11	0.40
SF-12 (MCS)	38.79±9.24	36.99±9.84	0.002
Extension (degrees)	37.28 ±2.92	37.35 ±2.93	0.68
Flexion (degrees)	37.21 ±3.22	37.18 ±3.46	0.89
Pronation (degrees)	79.16 ±7.54	79.36 ±7.3	0.39
Supination (degrees)	79.44 ±7.59	79.36 ±7.58	0.27
Grip strength (% of uninjured side)	72.31 ±4.42	72.54 ±4.47	0.40
***6 months***			
PRWE score	28.51 ±6.64	28.05±6.22	0.46
qDASH score	14.92 ±2.28	14.80 ±2.26	0.40
Mayo’s wrist score	20.87±6.51	21.51±6.51	0.16
SF-12 (PCS)	32.08 ±4.55	32.19 ±4.53	0.68
SF-12 (MCS)	38.30±8.16	39.33±8.48	0.04
Extension (degrees)	43.58 ±7.35	43.64 ±7.25	0.89
Flexion (degrees)	38.81 ±5.31	39.11 ±5.31	0.37
Pronation (degrees)	83.02 ±7.26	83.40 ±7.22	0.22
Supination (degrees)	83.11 ±7.08	82.62 ±7.55	0.71
Grip strength (% of uninjured side)	75.03 ±2.67	75.11 ±2.61	0.61
***1-year***			
PRWE score	10.31 ±3.45	10.44 ±3.27	0.54
qDASH score	11.90 ±2.74	12.20 ±2.69	0.07
Mayo’s wrist score	88.53 ±5.34	88.59 ±5.39	0.87
SF-12 (PCS)	37.96 ±2.15	37.73 ±2.15	0.09
SF-12 (MCS)	40.70±8.96	41.36±9.12	0.23
Extension (degrees)	55.20 ±10.38	55.71 ±10.16	0.42
Flexion (degrees)	51.44 ±5.30	51.67±4.92	0.47
Pronation (degrees)	87.12 ±3.90	86.98 ±4.11	0.58
Supination (degrees)	86.94 ±4.24	86.68 ±4.73	0.34
Grip strength (% of uninjured side)	77.87 ±4.60	77.77 ±4.71	0.72

P-value for continuous variable was calculated by independent t-test and Mann-Whitney U-test.

With regards to radiological outcomes there was no significant difference in the radiological parameters at the time of injury. In contrast, during the follow up periods, plating group had significant improvement in all the radiographic parameters in comparison to the no operative group (Table-III).

**Table-III:** Comparison of Radiologic Variables among Patients with Both Groups.

	Intraarticular fractures			Extraarticular fractures		

	Cast-Immobilization (283)	Volar Locking Plate (290)	p-value	Cast-Immobilization (246)	Volar Locking Plate (244)	p-value
***Radial Height (mm)***					
At time of injury	9.96±2.30	10.21±2.25	0.17	10.21±2.19	9.88±2.18	0.16
three months	9.99±2.20	9.81±2.30	0.34	10.03±2.20	9.78±2.25	0.27
one year	9.99±2.20	9.84±2.28	0.42	10.09±2.22	9.78±2.25	0.16
***Radial Inclination degrees***					
At time of injury	20.27±3.04	20.22±2.75	0.84	20.35±3.05	19.83±2.56	0.55
three months	19.94±2.99	19.80±2.35	0.53	16.43±5.25	21.57±3.12	0.001
one year	20.29±3.06	19.89±2.43	0.09	16.58±5.26	21.80±3.17	0.001
***Palmar tilt in degrees***					
At time of injury	2.58±1.69	2.73±1.57	0.24	2.65±1.65	2.86±1.46	0.13
three months	2.33±1.79	2.43±1.74	0.48	2.84±1.40	2.58±1.65	0.06
one year	2.33±1.79	2.43±1.74	0.48	2.84±1.40	2.58±1.65	0.06
***Articular Step off (mm)***				N/A	N/A	N/A
At time of injury	1.08±1.24	1.24±1.37	0.17			
three months	1.40±1.46	0.66±0.53	0.001			
one year	1.46±1.46	0.71±0.54	0.001			
***Ulnar Variance (mm)***					
At time of injury	0.64±0.52	0.66±0.65	0.65	0.62±0.62	0.42±0.43	0.001
three months	1.74±1.50	0.67±0.68	0.001	1.57±1.46	0.63±0.54	0.001
one year	1.42±1.38	0.60±0.60	0.001	1.47±1.43	0.60±0.51	0.001

A greater number of complications were noted in the cast group in comparison to the plating group (Table-IV). Similarly, malunion was significantly higher in the cast group as compared to the plating group. Subsequently, a greater number of corrective osteotomies were performed in the cast group. Also, the incidence of transient nerve palsy and CRPS was seen more prevalent in the cast group.

**Table-IV:** Complications and Additional Procedures.

Complications & Extra Procedures	Cast-Immobilization (529)	Volar Locking Plate (534)
Superficial infection	7	8
Transient nerve palsy	15	0
Carpal tunnel syndrome	7	11
Ulnar styloid pain	9	6
Nonspecific wrist pain	3	7
Extensor pollicis longus rupture	0	1
Plate impingement	0	2
Malunion	17	8
Complex Regional Pain Syndrome	23	11
Carpal tunnel decompression	2	1
Corrective Osteotomy	14	6
Plate Removal	0	3

Total	97	64

## DISCUSSION

DRF accounts for 18% of orthopaedic injuries in the elderly^17^ which can lead to significant disability.^18^,^19^ DRFs are treated either conservatively or surgically via open reduction and internal fixation (ORIF). This RCT showed that there is no significant difference in PRWE at one year; in patients aged over 60 years with dorsally, displaced DRFs when treated by cast immobilization or ORIF. Also, in most secondary outcomes, no difference was found at one year. However, a little but significant difference in favor of ORIF was found at three months in the PRWE, qDASH score and Mayo’s wrist score.

This difference may suggest a faster initial recovery, but may not be clinically relevant, because this difference is below the MCID. At the end of follow-up the radiological parameters were significantly better in the ORIF group and the number of complications higher in the non-operative group. These two differences seem to be related and may be clinically relevant. The two groups differ mostly in the number of malunion, CRPS and the number of secondary corrective osteotomy. Malunion and a secondary osteotomy goes hand in hand and may be avoided by an anatomical reduction and strong fixation in the ORIF group.

The clinical outcomes of our trial are consistent with couple of prospective RCT conducted by Arora et al.^7^,^11^ and Bartl et al.^8^ In these trials, the authors compared the clinical outcomes of DRF treated by ORIF or cast immobilization. The study population in Arora et al. included both extra-articular and intra-articular fractures,^11^ whereas Bartl et al. evaluated the outcomes in intra-articular fractures.^8^ Neither of the trials showed superiority of one treatment over other when clinical outcomes were evaluated at six or twelve months. Young and Ryan reported the outcome of nonoperative treatment of DRF in low demand elderly patients.^20^ The mean age in their study was 72 years and the average follow-up period was 34 months. They found no correlation between radiographic outcome and functional outcome. In their series, 60% of patients with intra-articular fracture progressed to radiocarpal and radioulnar arthritis and 33% of these patients had unsatisfactory outcome. Fifty-six percent of the patients had obvious clinical deformities but none of them were dissatisfied with the appearance of the wrist.^20^

**Fig.2:**
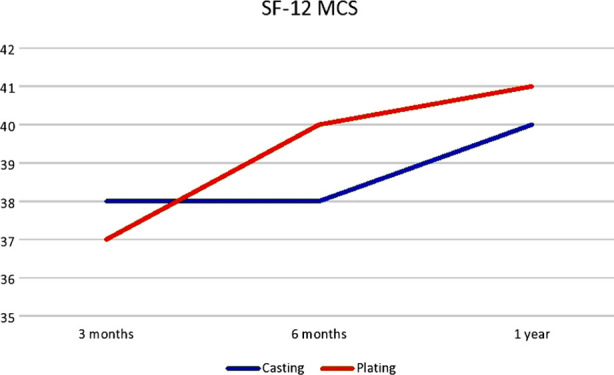
Patient Satisfaction (SF-12) Mental Component during the follow-up period.

Likewise, Egol et al. conducted a retrospective study comparing the outcomes of displaced DRFs treated operatively or non-operatively in patients over the age of sixty-five years old.^21^ They found no significant difference between the groups with regards to DASH or pain scores during the follow-up period. The radiographic parameters showed better results in the operative group and there was no difference in terms of complications between the two groups.^21^ Recently, Chung et al. conducted a multicenter RCT comprising 296 participants on DRF treatment in patients aged 60 years and older.^22^ They had four treatment arms in their trial for managing DRFs, which were volar locking plate, percutaneous pinning, external fixation, and casting. They reported significant number of complications in the casting group as compared to other groups.^22^ They also suggested that accurate restoration of wrist anatomy is not related with better patient outcomes in older patients at 12 months follow up.^22^ Likewise in our study poor radiographic outcomes did not transform into poor patient satisfaction in the casting group as shown in Fig.2.

Despite these publications, our study is showing, that even if there is no clinical difference at the end of follow-up, and the radiological and clinical outcome seems not to be related one to another, there are cases in which a malunion is leading to a corrective osteotomy. In these cases, the malunion may be perceived so troublesome to justify another surgery. It is necessary that future studies, try to filter out these factors to improve patient outcome, on avoiding these complications.

### Limitations:

Our RCT has limitations; the surgeon and patient were not blinded to treatment groups. An independent team collected outcome measures, avoiding ascertainment bias. Also, patients completed clinical outcome questionnaires at each follow-up without staff input. Second, the 12-month follow-up may be too short to evaluate long-term complications like radiocarpal or radioulnar osteoarthritis. Symptoms may not always accompany radiographic arthritis.^23^ Finally, we would like to disclose that though the approval of study was granted to us in 2015 though retrospective registration of trials in public registry in orthopedics was made mandatory from January 2018.^24^

### Strengths:

The study followed CONSORT,^25^ our study was well-powered, and most patients completed the final follow-up, ensuring its validity and generalizability. Our clinical outcomes were evaluated using validated patient-reported measures. The treatment groups had similar baseline characteristics.

## CONCLUSION

The results of the trial have shown that ORIF and cast immobilization are equally effective in achieving patient satisfaction, patient reported and clinical outcomes at intermediate and final follow-up. However, casting seems to have more complications.
